# The Survival Condition and Immunoregulatory Function of Adipose Stromal Vascular Fraction (SVF) in the Early Stage of Nonvascularized Adipose Transplantation

**DOI:** 10.1371/journal.pone.0080364

**Published:** 2013-11-18

**Authors:** Ziqing Dong, Zhangsong Peng, Qiang Chang, Feng Lu

**Affiliations:** Department of Plastic and Cosmetic Surgery, Nanfang Hospital, Southern Medical University, Guang Zhou, Guang Dong, P.R , China; Rutgers - New Jersey Medical School, United States of America

## Abstract

**Introduction:**

Adipose tissue transplantation is one of the standard procedures for soft-tissue augmentation, reconstruction, and rejuvenation. However, it is unknown as to how the graft survives after transplantation. We thus seek out to investigate the roles of different cellular components in the survival of graft.

**Materials & Methods:**

The ratios of stromal vascular fraction (SVF) cellular components from human adipose tissue were evaluated using flow cytometry. Human liposuction aspirates that were either mixed with marked SVF cells or PBS were transplanted into nude mice. The graft was harvested and stained on days 1,4,7 and 14. The inflammation level of both SVF group and Fat-only group were also evaluated.

**Results:**

Flow cytometric analysis showed SVF cells mainly contained blood-derived cells, adipose-derived stromal cells (ASCs), and endothelial cells. Our study revealed that most cells are susceptible to death after transplantation, although CD34+ ASCs can remain viable for 14 days. Notably, we found that ASCs migrated to the peripheral edge of the graft. Moreover, the RT-PCR and the immuno-fluorescence examination revealed that although the SVF did not reduce the number of infiltrating immune cells (macrophages) in the transplant, it does have an immunoregulatory function of up-regulating the expression of CD163 and CD206 and down-regulating that of IL-1β, IL-6.

**Conclusions:**

Our study suggests that the survival of adipose tissue after nonvascularized adipose transplantation may be due to the ASCs in SVF cells. Additionally, the immunoregulatory function of SVF cells may be indirectly contributing to the remolding of adipose transplant, which may lead to SVF-enriched adipose transplantation.

## Introduction

Free-fat grafting has become one of the standard procedures for soft-tissue augmentation, reconstruction, and rejuvenation. It is safe and easy to perform, does not leave scars on donors or recipient sites, and it does not result in cross-infection or foreign-body reaction [1–3]. However, the major limitation of free-fat grafting is the replacement of fibrotic tissues resulting from fat absorption and fat necrosis. This may also lead to the shrinkage of adipose tissues over time [4]. 

Adipose stromal-vascular fraction (SVF) contains adipose-derived stromal cells (ASCs) [5,6]. According to previous studies, adipose SVF cells can increase the survival of ischemic tissues like ischemic hindlimbs, random skin flaps, and acutely infarcted myocardium [7–9]. SVF cells may improve tissue outcomes by increasing vascularity and the secretion of growth factors which improve tissue survival. Blood vascular endothelial cells (ECs) in SVF cells also play a role in angiogenesis and are incorporated into new vessels through dynamic reassemblies [10]. Some reports have also demonstrated the anti-inflammatory role of SVF cells in changing the micro-environment [11,12]. However, the survival condition and the mechanisms of both mature adipocytes and SVF cells after transplantation have not been well studied.

The goal of the present study was to clarify the survival condition and cellular events after nonvascularized adipose tissue transplantation. Therefore, we focused on the early stage (1 to 14 days) after grafting, analyzed the viability of each cellular component of adipose tissue in vitro, and investigated the immunoregulatory capacity of SVF cells in the fat graft.

## Methods

### Ethics Statement

To obtain SVF cells, human adipose tissue collection and cell harvests were approved by the Southern Medical University human subject board (IRB approval no.15402). All patients and healthy subjects received an explanation about the scope of the study, such as objectives, procedures and potential risks, and signed an informed consent statement before inclusion in the study.

The animal experimental protocols were approved by the Southern Medical University Laboratory Animal Administration Committee and performed according to the Southern Medical University Guidelines for Animal Experimentation. All efforts were made to minimize suffering.

### Human Cell Isolation

Abdominal human liposuction aspirates were obtained from seven healthy females who have no systemic disease. Their body mass indexes (BMI) were between 23.5 and 25.2 kg m^-2^ with a mean age of 32.4±4.8 years. Lipoaspirates were washed with phosphate buffered saline (PBS) and then centrifuged at 430 g for 5 minutes. After oil was removed, the lipid phase of the lipoaspirate from the top of the conical tube was harvested and divided into two parts: one for cell culture and the other for ensuring transplantation. The adipose tissue was digested in 0.075% collagenase (Wako Pure Chemical Industries, Ltd., Osaka, Japan) at 37°C for 30 minutes on a shaker. Mature adipocytes and connective tissue were separated from pellets by centrifugation (800 g for 10 minutes) and then discarded. The pellets were re-suspended in phosphate-buffered saline and filtered through a 200μm mesh followed by centrifugation (800 g for 10 minutes) to spin down stromal vascular fraction cell pellets. 

### Flow Cytometry

Characterization of SVF was performed by fluorescence-activated cell sorting (FACS) analysis. The staining of cells with fluorescein isothiocyanate–conjugated anti-human mouse antibodies included anti-CD31, anti-CD34, and anti-CD45 (BD Biosciences, San Jose, California, USA). Multicolor flow cytometry was performed with an LSR II (BD Biosciences), and cell composition percentages were calculated according to data of surface marker expression profiles.

### Hoechst 33342 Staining of SVF Cells

SVF cells were labeled with Hoechst 33342 (Sigma, Missouri, USA) according to the manufacturer’s recommendations. Cells in suspension were then immediately incubated with Hoechst 33342 at a concentration of 20 μg/ml in PBS for 15 minutes at 37°C and then washed thrice.

### Animal Models

Animals were cared for in accordance with our institutional guidelines. 28 nude mice of Health SPF level (provided by experimental animal center, Nanfang Medical University), weighing 15-18g at age 4-6 weeks (gender unregarded) were used as free fat transplantation models. 0.5ml of prepared human lipoaspirates were mixed with 5×10^6^ marked SVF cells suspended with 10ul PBS. The mixture was injected into the subcutaneous tissues on the left flank of the nude mouse using a 1 ml syringe with standardized blunt tipped 14 gauge infiltration cannula (SVF group). 0.5ml of fat mixed with 10ul PBS was injected into the right flank of the nude mouse as the fat only group. At 1, 4, 7 and 14 days after fat transplantation, the grafts were excised with CO_2_ and analyzed as described below. No animals died in this study.

### Whole-Mount Staining of Grafted Fat Tissue

Visualization of Grafted Fat Tissue was performed using the procedure of Hitomi Eto et al [13]. Accordingly, the adipose tissue was cut into 0.5-1 mm pieces and incubated with the following reagents for 60 minutes: BODIPY 558/568-conjugated phalloidin (Molecular Probes, Eugene, Ore.) to stain adipocytes, Alexa Fluor 488-conjugated isolectin GS-IB4 (Molecular Probes) to stain endothelial cells, Hoechst 33342 to stain all nuclei (only for co-stain with F4/80), Alexa Fluor 488-conjugated anti-mouse F4/80 (Biolegend, California, USA), or Alexa Fluor 488-conjugated anti-human CD34 (Biolegend, California, USA). The sample was then washed and observed directly with a confocal microscope system (Leica TCS SP2; Leica Microsystems GmbH, Wetzlar, Germany). Eight to ten images acquired with every 3-μm interval were used for reconstructing three-dimensional images.

Hoechst positive cells in SVF group were counted, and the total percentage of surviving SVF cells was referred to as the quantity of mixed cells (5×10^6^ cells). The survival of different cell lineage was measured by counting double-positive for Hoechst and lineage specific markers. The number of each cell population per unit volume was then calculated. Four field images of each sample were analyzed.

### Quantitative real-time PCR

The total RNA was extracted from both fat graft and SVF cells-assisted fat graft using the RNeasy kit (Qiagen, Germany). DNase treatment was conducted during the RNA extraction using RNase-Free DNase Set (Qiagen, Germany) to avoid genomic DNA contamination. Furthermore, no template control (NTC) was run in each experiment. cDNA was synthesized using multiscribe reverse transcriptase (Applied Biosystems, CA, USA). Quantitative RT-PCR for each gene was performed using the TaqMan method (50 °C for 2 min, 95 °C for 10 min, and 40 cycles of 95 °C for 15 sec and 60 °C for 1 min) with Premade Primer sets (Applied Biosystems, CA, USA). We used the standard curve method for the quantitative analysis of quantitative RT-PCR. The standard curve was drawn from the Ct values of the samples. The expression levels of each sample were determined using the standard curve. Reverse-transcriptase polymerase chain reaction was conducted with primers designed for this study (Table 1). The relative abundance of the transcripts was optimized based on the expression of GAPDH mRNA.

**Table 1:** Primer Sequences for Real-Time Reverse-Transcriptase Polymerase Chain Reaction.

**Gene(mouse)**	**Forward**	**Reverse**
CD163	5’-TGCCAAACCGTGGAGTCACA-3’	5’-CGCTGAATCTGTCGTCGCTT-3’
CD206	5’-TCCCTGCCTGTTTCTCCAACCA-3’	5’-TAAGCTTCGGCTCGTCAGCA-3’
IL-1β	5’-TGCACTACAGGCTCCGAGAT-3’	5’-GTGGGTGTGCCGTCTTTCAT-3’
IL-6	5’-ACAACCACGGCCTTCCCTACTT-3’	5’-AGCCTCCGACTTGTGAAGTGGT-3’
GAPDH	5’-GGCCTCCAAGGAGTAAGAAA-3’	5’-RGCCCCTCCTGTTATTATGG-3’

### Statistical Analysis

The data is expressed as mean ± SEM. A repeated measures analysis of variance was used to analyze the results. Furthermore, if statistical significance was reached, paired Student’s t-test of two groups in one time point and one-way analysis of variance (ANOVA) of one group in four time points was performed. A value of P < 0.05 was considered significant.

## Results

### SVF Cell characterization

**Figure 1:**
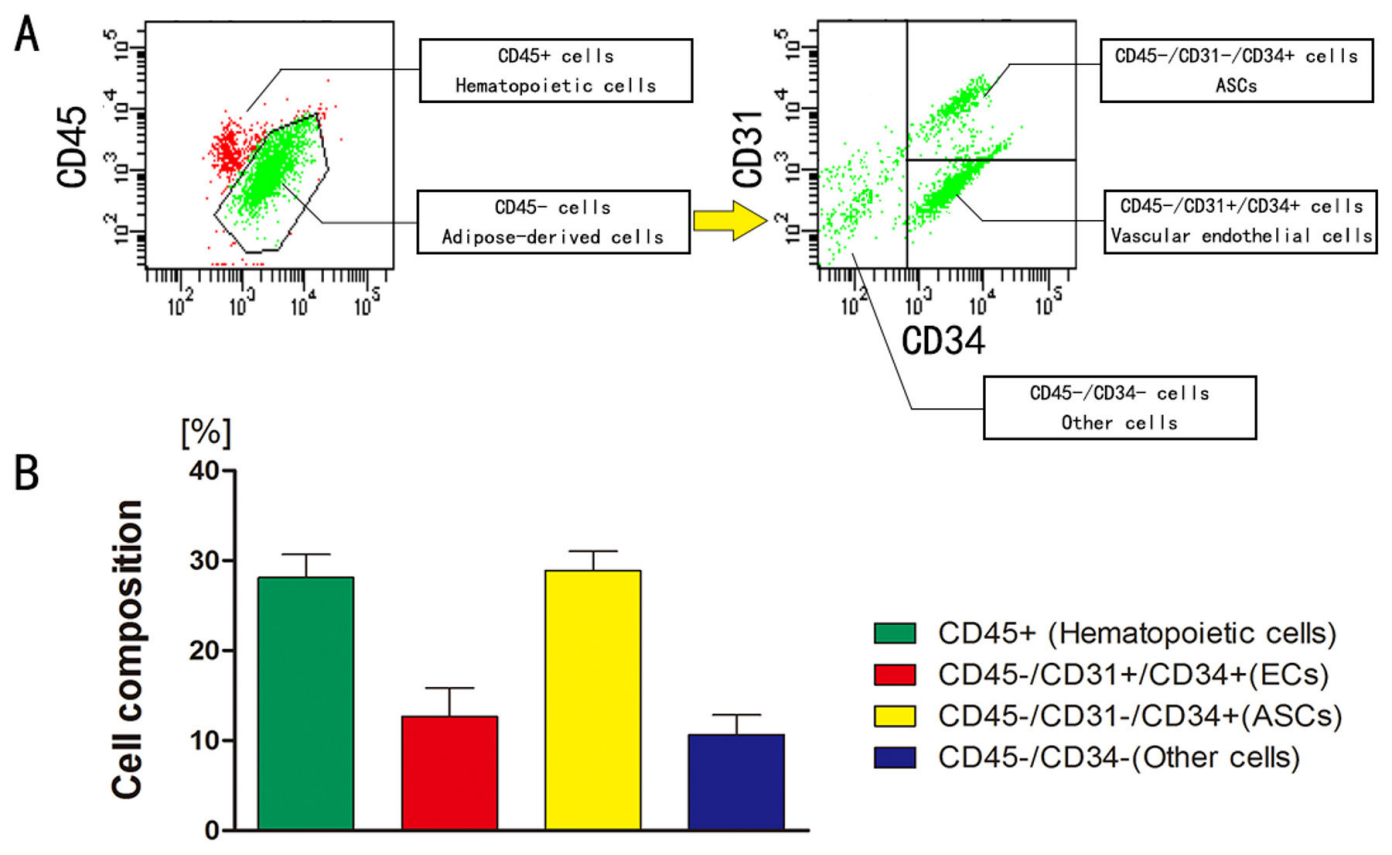
Flow cytometric analysis of SVF cells derived from human aspirated adipose tissue. A: Representative plotted data. CD45+ cells were hematopoietic cells (derived mainly from peripheral blood), whereas CD45- cells were adipose tissue-derived cells. CD45-/CD31-/CD34+ cells, CD45-/CD31+/CD34+ cells, and CD45-/CD34- cells were regarded as ASCs, ECs, and other cells (such as fibroblasts and mural cells) respectively.

Freshly isolated SVF from human aspirated adipose tissue contains all types of cells in adipose tissue except mature adipocytes. Based on flow cytometric analysis; SVF cells contained 28.1±2.4% CD45+ cells (blood-derived cells), 28.9±2.0 % ASCs (CD45-/CD31-/CD34+), 12.7±2.9 % endothelial cells (CD45-/CD31+/CD34+) and 10.7±2.1 % other cells (fibroblasts and mural cells) (n = 7) (Figure 1).

### Viability of Adipocytes in Transplanted Tissue

**Figure 2:**
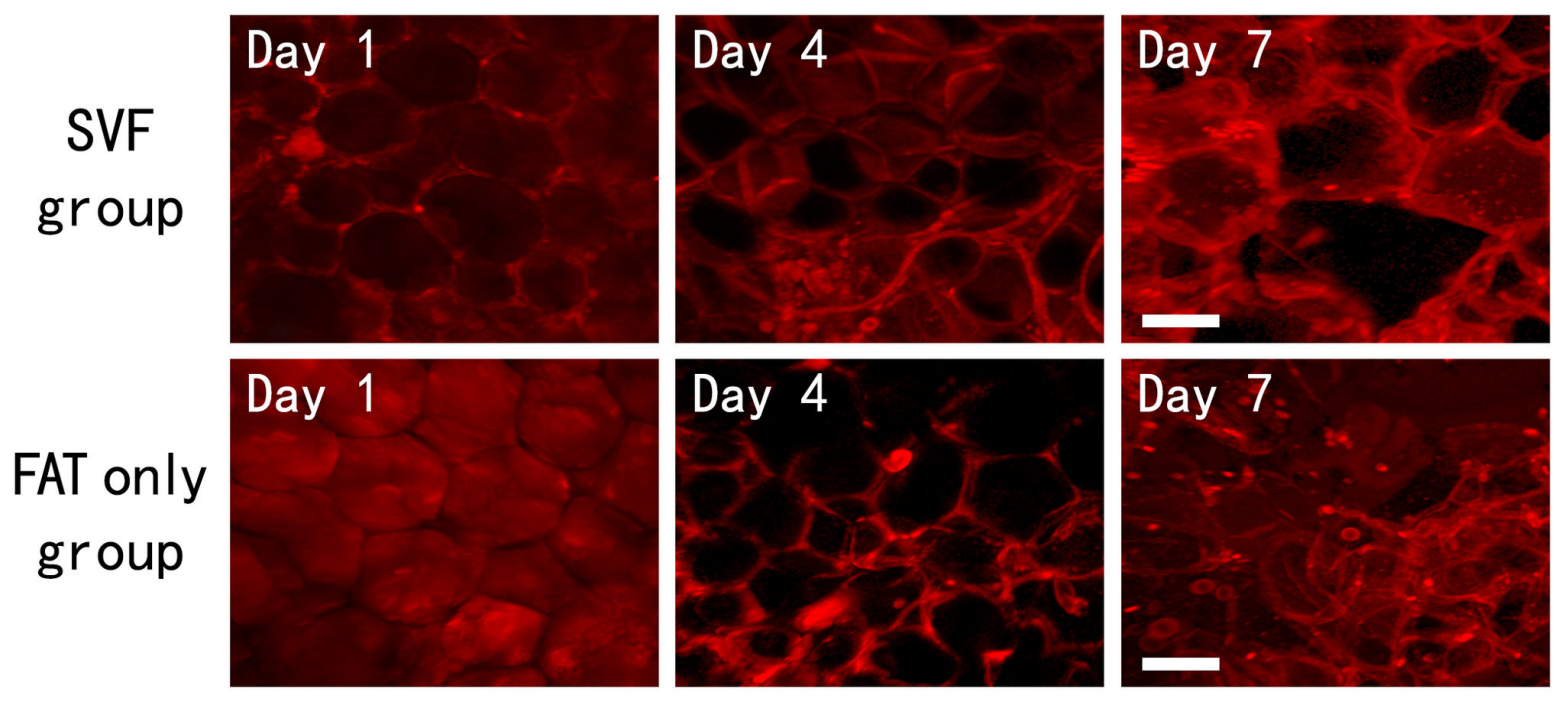
Whole-Mount Staining of adipocytes within the graft. The cultured adipose tissue was fixed at each time point and stained for phalloidin (viable adipocytes; red). Scale bars=50μm.

The graft was harvested (day 1, 4, and 7) and immuno-stained in order for phalloidin to stain mature adipocytes. On day 1, most adipocytes in the graft could still maintain the round shape. The number of irregularly shaped adipocytes significantly increased by day 4, and nearly all adipocytes lost their round shape by day 7 (Figure 2) except those on the peripheral edge. This phenomenon appeared in both groups in our study and suggests that the majority of mature adipocytes died after the transplantation. 

### The total retention of marked SVF cells

**Figure 3:**
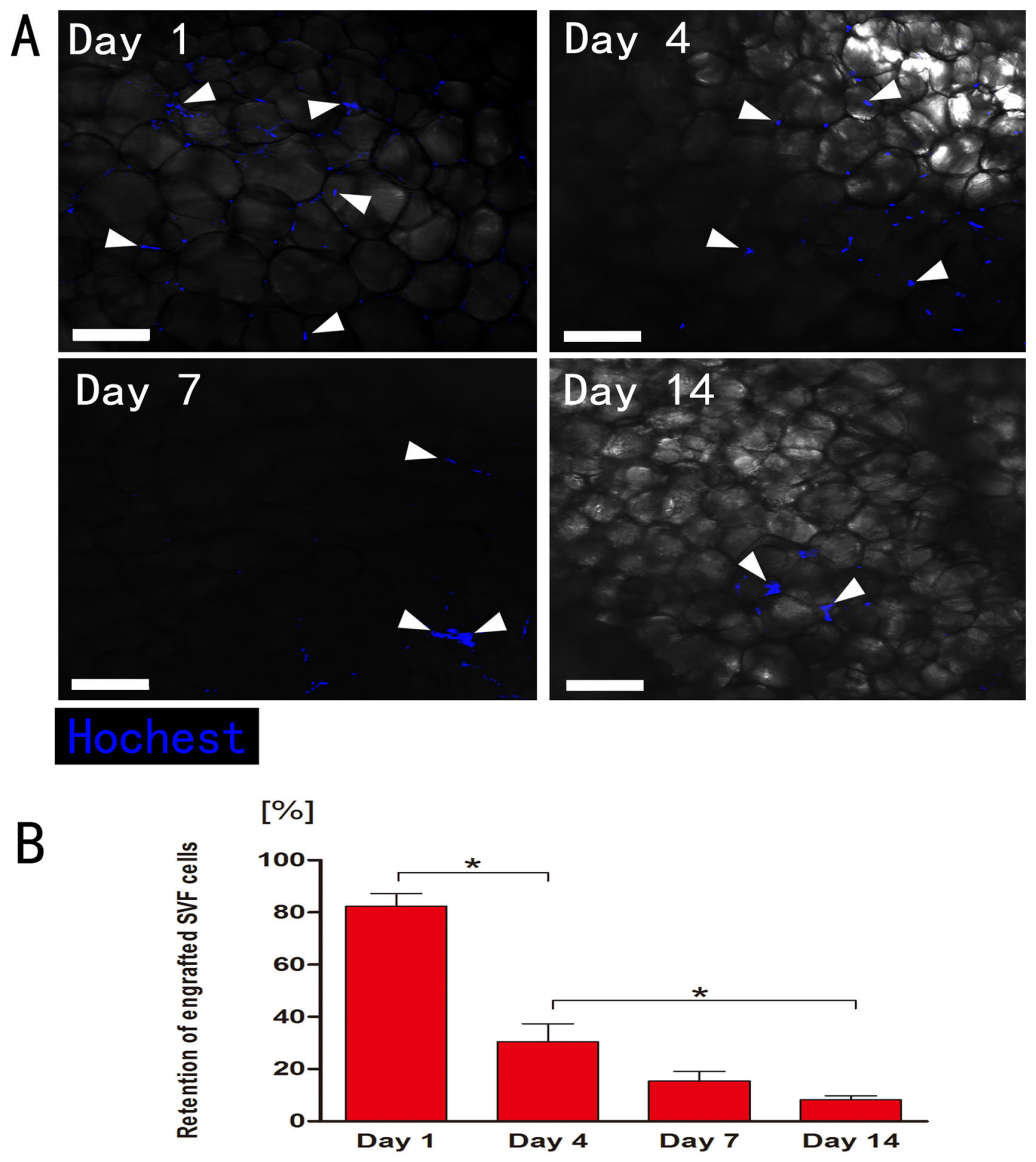
Immunohistology of the surviving marked SVF cells in graft between 1-14 days post-transplantation. A: Cells were detected at all time-points by Hoechst 33342 fluorescent (blue) staining (white arrowheads). Scale bars = 100μm. B: Quantification of surviving (Hoechst-positive) SVF cells (n = 7). The total surviving rate of SVF cells decreased sharply on day 1, followed by a further decrease over time. Statistical analysis was performed among the four time points (*p< 0.05).

The graft from SVF group was extirpated (day 1, 4, 7, or 14) and prepared for whole-mount staining. Detection of Hoechst 33342 throughout the experiment (day 1, 4, 7, or 14) revealed no positive Hoechst-signal in Fat-only group as expected. Conversely, the presence of Hoechst positive cells was demonstrated at all the time-points analyzed in all transplants from SVF group (n=7) although their number decreased over time (Figure 3, A). Quantification of marked SVF cells indicated the retention decreased from day 1 (82.3±12.2%) after transplantation, sharply at day 4 (30.4±17.0%), and further at later time-points (day 7: 15.4±9.1%, day 14: 8.2±3.7%) (Figure 3, B).

### ECs in SVF may promote angiogenesis through dynamic reassembly

**Figure 4:**
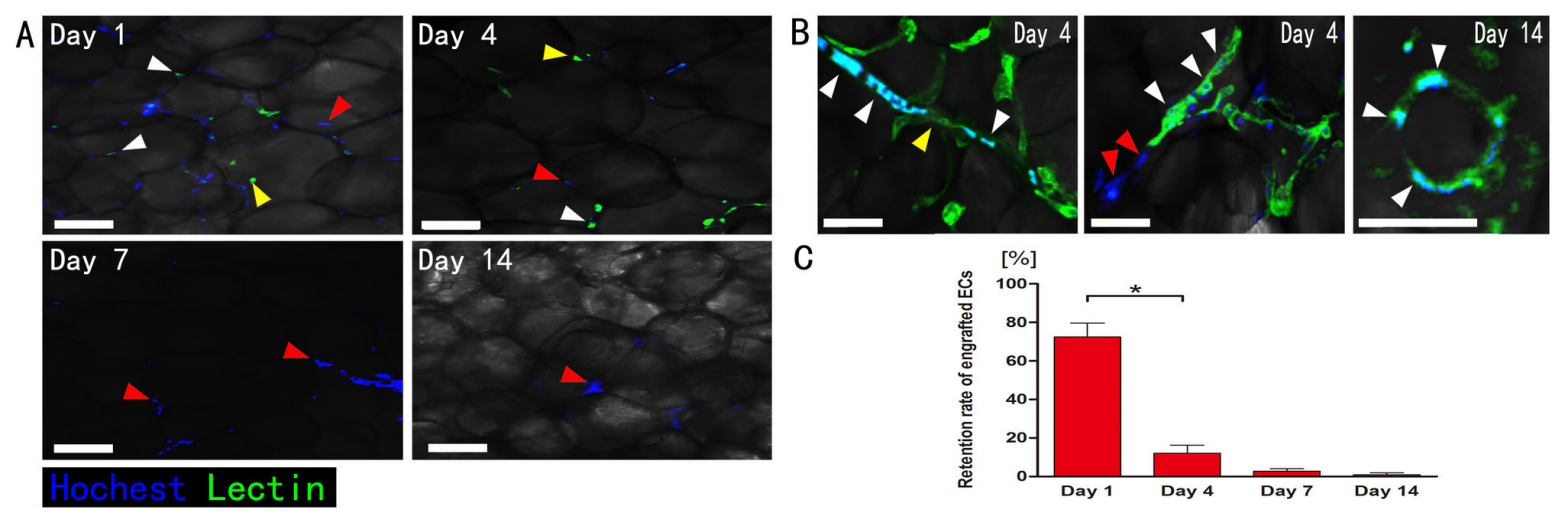
Immunohistology of ECs in the graft. A: Lectin and Hoechst double positive cells were regarded as marked ECs (white arrowheads), otherwise (Lectin-positive only) as which were from the graft (yellow arrowheads), and Hoechst-positive only cells were identified as marked non-endothelial cells (red arrowheads). Scale bars = 50μm. B: A few marked ECs (white arrowheads), un-marked ECs (yellow arrowheads) (left) and those non-endothelial SVF cells (red arrowheads) could form cell cords (middle) on day 4. Scale bars = 20μm. Moreover, ECs from marked SVF cells formed blood vessel structure (right). Scale bars = 10μm. C: Quantification of surviving (Hoechst/Lectin-double positive) marked ECs (n = 7). The retention ratios for ECs fell off sharply from day 1 to day 4, which decreased further at day 14 (*p< 0.05).

The SVF cells within the graft of SVF group could be divided into two parts, one was the Hoechst marked SVF and the other was un-marked SVF derived from the tissue. Immunostaining with Lectin depicts survival condition of all the ECs within SVF cells. ECs positive for Hoechst were considered as the ones from marked SVF (white arrowheads) (Figure 4, A), while those negative for Hoechst were from the graft (yellow arrowheads). The number of all the ECs decreased over time after transplantation. Remarkably, ECs are hardly detectable by the seventh day after transplantation, and the surviving marked SVF cells were almost non-endothelial cells (red arrowheads) (Figure 4, A). 

Interestingly, we observed a few ECs (from marked SVF and un-marked SVF) and non-endothelial SVF cells could form cell cords (Figure 4, B, middle) on day 4. Additionally, ECs also formed the blood vessel lumen structure (Figure 4, B, right).

After counting the number of Hoechst-positive ECs, we calculated the retention of marked ECs with the percentage of the FACS data on SVF cells isolated from adipose tissue. SVF cells were composed of 12.7% ECs (on average), which means there were 5×10^6^×12.7% ECs in each graft. The retention could be calculated by using the ratio of surviving marked ECs density to the previously marked ECs density for each graft. The retention ratios for ECs went down sharply from day 1 (71.7±19.6%) to day 4 (12.1±10.1%), and decreased further at day 14 (0.9±2.3%) (Figure 4, C).

### ASCs in SVF can survive the ischemic condition and migrate to the peripheral edge

**Figure 5:**
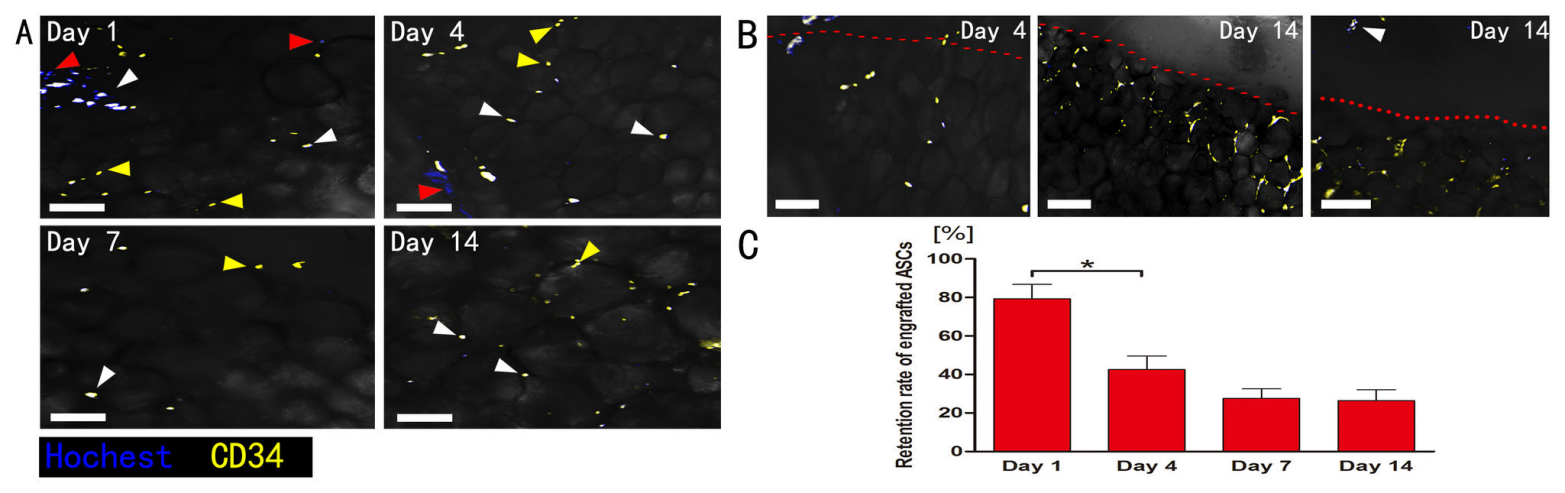
Immunohistology of the ASCs. A: The number of ASCs (CD34 positive) cells from both marked SVF cells (white arrowheads) and un-marked (yellow arrowheads) decreased over time. Almost all the survived SVF cells were CD34 positive by day 7, non-CD34 positive SVF cells (red arrowheads) can’t be detected after day 4. B: The CD34 positive cells migrated to the peripheral edge (red dotted line) over time, some of them even crossed the peripheral edge (white arrowhead). Scale bars = 50μm. C: Quantification of surviving marked ASCs (n = 7). C: The retention of marked ASCs significantly fell from day 1 to day 4 then made no significantly change till day 14 (*p< 0.05).

The number of CD34 positive cells from SVF decreased over time. However, almost all the survived SVF cells were CD34 positive by day 7. The non-CD34 positive cells (red arrowheads) could not be detected after day 4 (Figure 5, A).

Because ECs are also CD34 positive, we counted the number of CD34 and Hoechst double positive cells and then subtracted the number of marked ECs from the same sample as the number of ASCs. Using this method, we calculated the retention ratios of ASCs. By quantification, the retention of ASCs significantly decreased from day 1 (79.3±18.5%) to day 4 (42.5±17.5%), but there was no significant decrease after day 4 (day 7: 27.6±12.0%, day 14: 26.5±13.8%) (Figure 5, C).

The location of CD34 positive cells demonstrated a definitive change over time. During the initial four days, the CD34 cells distributed evenly throughout the graft. By day 14 the CD34 cell density increased from the peripheral region (red dotted line). Some even crossed the peripheral edge (white arrowhead). There was no significant decrease of the CD34 cell number after day 4, suggesting these cells might have migrated to the peripheral edge (Figure 5, B).

### SVF cells can regulate immune response in the early stage of transplantation

**Figure 6:**
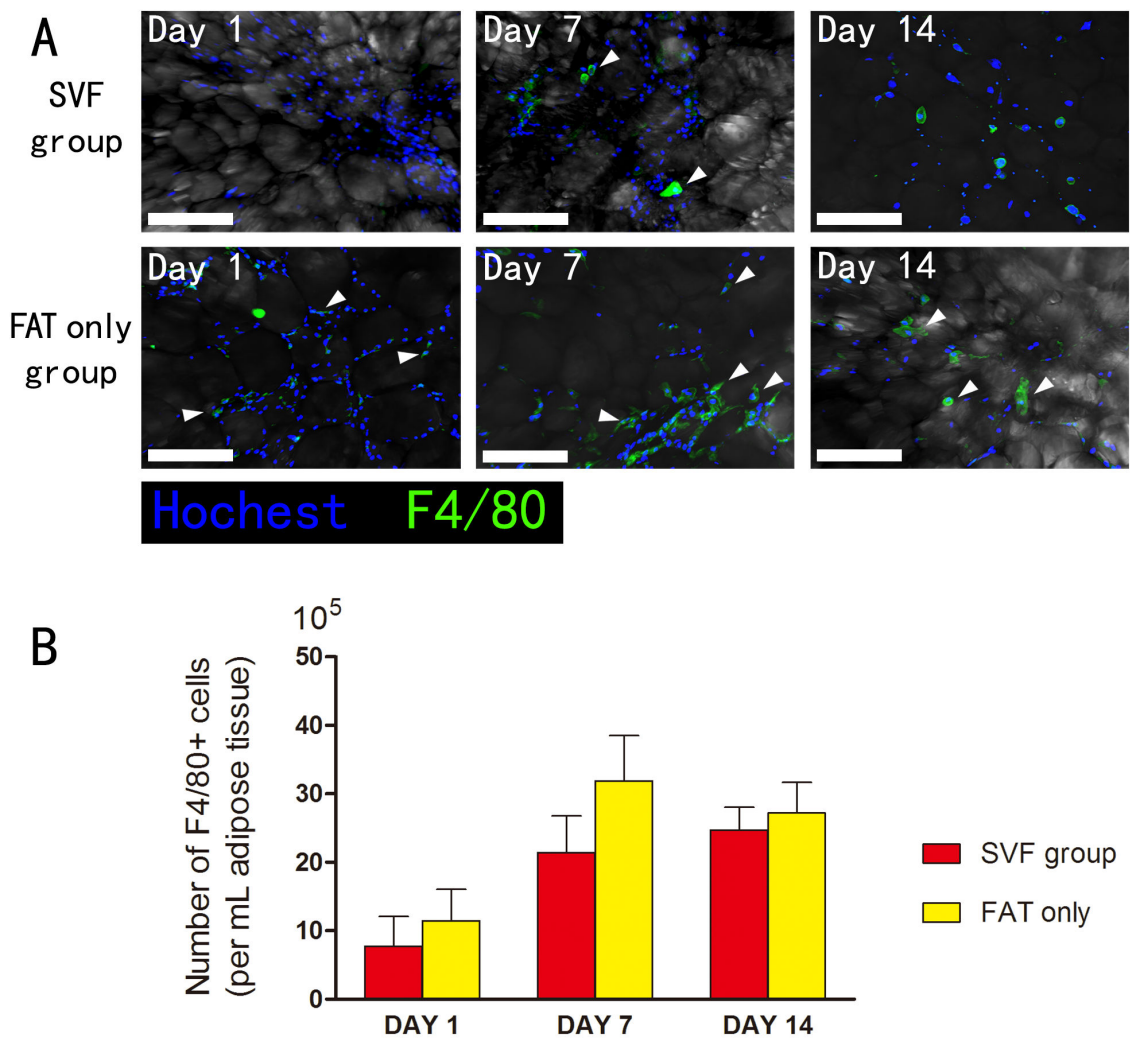
Immunohistology of the macrophages in the graft. A: Immunostain demonstrated the infiltration of macrophages (F4/80 positive cells) (white arrowheads) in both groups. Scale bars = 50μm. B: Quantification of number of macrophages in the transplants increased from day 1 to day 7 in both groups, then it fell slightly on day 14. And the numbers of infiltrated macrophages (n = 7) had no significant difference between the SVF group and Fat-only group (*p< 0.05).

Immunostain demonstrated the infiltration of macrophages (F4/80 positive cells) (white arrowheads) in both groups (Figure 6, A). The number of macrophages in the grafts increased from day 1 to day 14 in the SVF group. As for the fat only group, the number increased from day 1 to day 7, and then slightly decreased at day 14. Statistical analysis was performed between the two groups at each time point (*p < 0.05). Interestingly, the numbers of infiltrated macrophages had no significant difference between the SVF group and fat only group (Figure 6, B). 

**Figure 7:**
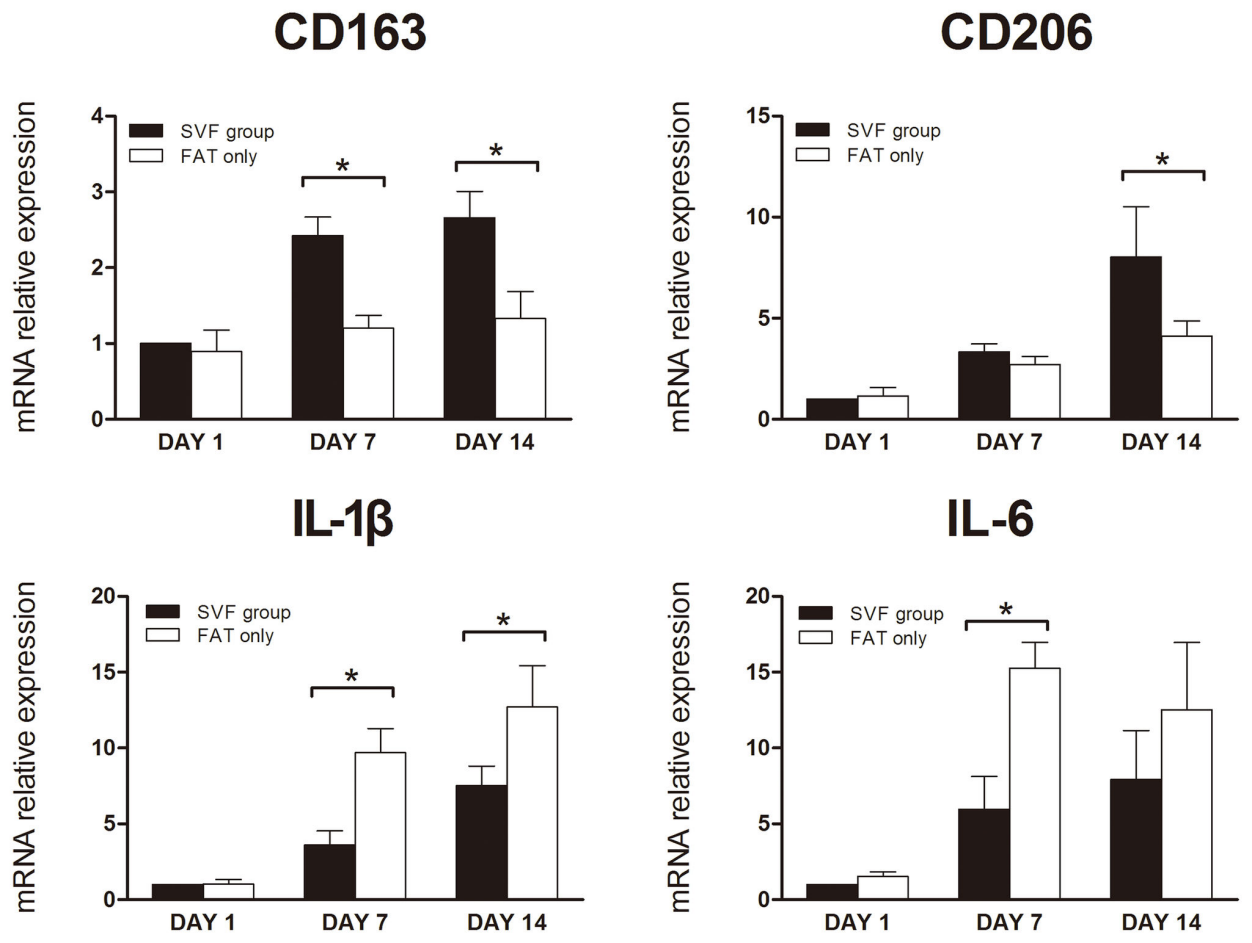
The mRNA expression in the transplants. The expression of CD163 on day 7 and day 14, and CD206 on day 14 was significantly higher in SVF group. However, the expression of IL-1β was lower in SVF group on day 7 and day 14. IL-6 expression was lower in SVF group at day 14 too. All groups n= 6 (*p< 0.05).

The tissue mRNA expression of CD163 and CD206 from both groups increased from day 1 to day 14. Statistical analysis showed that SVF group had a higher expression level of CD163 (day 7 and day 14) and CD206 (day 14) (*p < 0.05). As for the IL-1β and IL-6, all the expression levels increased from day 1 to day 14, except the IL-6 level of the fat only group which decreased slightly from day 7 to day 14. Interestingly, statistical analysis showed that the SVF group had a lower expression level of IL-1β (day 7 and day 14) and IL-6 (day 7) (*p < 0.05) (Figure 7).

## Discussion

**Figure 8:**
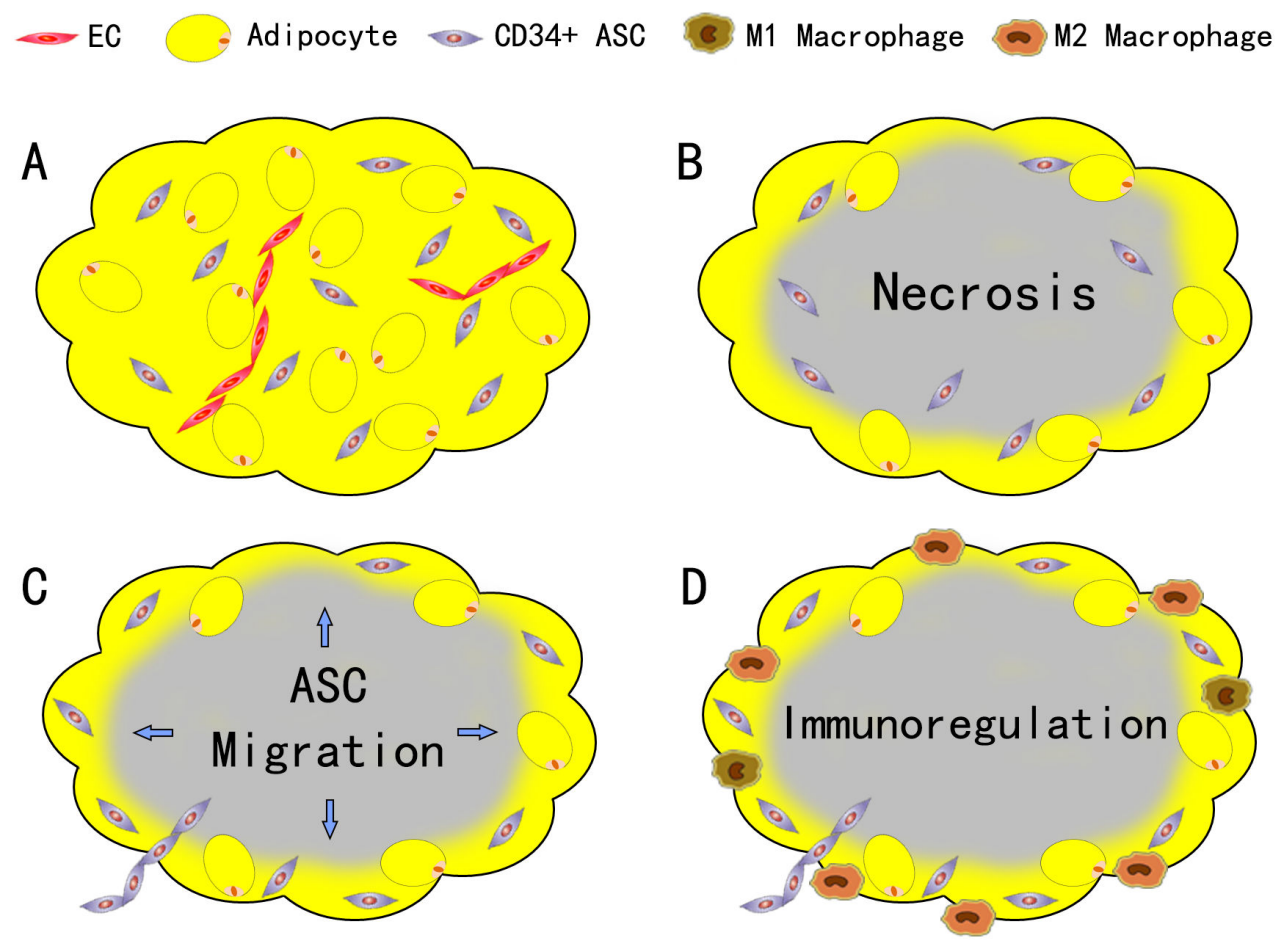
Conclusion of the findings. A: At the early stage of transplantation, most mature adipocytes and ECs necrosis during the ischemia, only the CD34+ ASCs could survive. B: Part of the survived ASCs and ECs could incorporate into the vascular structures. C: Survived ASCs would migrate to the edge of the graft. D: SVF cells did not aid in reduction of the immune cells (macrophages) infiltration, but showed an immunoregulatory capacity of up-regulating the expression of CD163 and CD206 and down-regulating IL-1β and IL-6 expression.

Several studies have focused on the therapeutical effect of SVF cells and ASCs in wound healing and ischemic diseases [7–9]. SVF and ASCs could improve tissue outcomes by increasing vascularity and increasing the secretion of growth factors that improves tissue survival. YOSHIMURA first reported that the assisting of SVF could enhance angiogenesis and improve the survival rate of graft [14]. In a recent in vitro study of showed that ECs and other cells in the graft died after the transplantation, only ASCs survived [15]. In this study, we use the nude mice model to investigate the fate of different cell lineages in SVF cells and their immunoregulatory function in the early stages after nonvascularized adipose transplantation. Our findings can be summarized as follows: 1) most mature adipocytes and ECs necrosis during the ischemia at the early stage of transplantation, only the CD34+ ASCs survived; 2) part of the survived ASCs and ECs could incorporate into the vascular structures; 3) survived ASCs could migrate to the edge of the graft; 4) SVF cells did not reduce the immune cells (macrophages) infiltration, but showed an immunoregulatory capacity of up-regulating the expression of CD163 and CD206 and down-regulating IL-1β and IL-6 expression (Figure 8).

In the early stages of adipose transplantation, inadequate blood supply would result in acute hypoxia of the implanted adipose tissues. In an acute ischemia model, a significant decrease in the fat pad pO_2_ was seen after ligating its feeding vessels arising from the femoral artery [16]. Nonvascularized adipose transplants obtain nutrients and oxygen through plasmatic diffusion from surrounding tissues until vascularization is restored. In Figure 2, the phalloidin staining showed that the majority of transplanted mature adipocytes died under ischemic condition within seven days regardless of the addition of SVF cells. Thus, the results do not support the cell survival theory. The regeneration of transplants may play the key role after transplantation. Moreover, the increase of the SVF cell density does not result in a better survival of mature adipocytes in our study, though it has been reported that SVF could increase the vessel density of the graft [14].

To further investigate the survival of SVF cells in adipose grafts, we used marked SVF cells mixed with human adipose tissue to simulate the normal condition of SVF cells in nonvascularized adipose transplants. Unexpectedly, in our study, the total survival rate of SVF cells is only 8.2% on day 14, suggesting that most SVF cells died under ischemic condition. The Lectin staining also showed ECs do not survive the ischemia for over four days. In the study of Y.J. Koh et al., ECs from SVF play a key role in angiogenesis by incorporating itself into new vessels through dynamic reassembly [10]. In vitro and in vivo studies have demonstrated the differentiation capacity of SVF into endothelial [17] and smooth muscle cells [18].. In our study, although the SVF cells especially ECs can form cell cords and blood vessel lumen in the early stages, they are very few in number. On the other hand, most newly formed functional vessels in the graft are from the host. 

In this study, we detected that only CD34+ cells from SVF but mature adipocytes could survive the ischemia after transplantation. These CD34+ cells are mainly located near the blood vessels in the adipose tissue [19]. Though it has been proved that the CD34+ ASCs could improve angiogenesis through secreting growth factors [20] , the CD34+ ASCs may have other role during grafting. Some data indicate that CD34+ cells are more proliferative than CD34- cells [21]. Another study showed that adipose stem cells are contained in the CD34+ cell population, and they are able to produce lipid-droplet formation activity under adipogenic culture condition [22]. Taken together, these evidences suggested that CD34+ ASCs might enhance the retention rate of the graft by neo-adipogenesis after transplant. 

We also found that the number of ASCs decreased sharply from day 1 to day 4 after transplantation, and the number remained almost unchanged until later phases of transplant. There were different sub-populations in ASCs. Ludovic Zimmerlin et al. suggested that the outer adventitial stromal ring (designated supra adventitial-adipose stromal cells, SA-ASC) was CD146-/α-SMA-/CD90+/CD34+/CD31-, and the CD146+/CD90+/CD34+/CD31- pericyte subset might be transitional between pericytes and SA-ASC [23]. The rapid decline in ASCs number may be accounted for by the selection of ischemia. Some specific cell populations that could survive the ischemia in the early stage of the transplantation will become the main functional cell population in the later stage. 

Migration of cells through tissue is well-described. A recent report by Scott T and colleagues demonstrated a novel finding of ASC migrating from the engraftment point in the flap across a wound interface into the surrounding native tissue when injecting rat ASCs into ischemic flaps [24]. In our study, the ASCs migrated from the engraftment position in the graft into the peripheral region, and some of them even crossed the edge. This unexpected finding suggests that these cells migrate from a hypoxic region to an oxygenated region, which raises the possibility that ASCs in the graft respond to a signal from an existing blood vessel lying in peripheral region of the transplant. This finding suggests that ASCs in the graft may join the adipose tissue proliferation process after transplantation through the migration mechanism.

Another potential role of SVF cells in tissue repair is immune regulation. It is known that macrophages originate from circulating monocytes. At the site of inflammation, recruited blood monocytes transmigrate across the endothelium and differentiate into macrophages. The two generally classified activation states of macrophages are M1 and M2 [25]. The classic M1 polarization state, defined as pro-inflammatory macrophages, initiates the inflammatory reaction by secreting high levels of pro-inflammatory cytokines (TNF-α, IL-6, IL-12, etc.). In contrast, the M2 state, or anti-inflammatory macrophages, secretes high levels of anti-inflammatory cytokines (IL-4, IL-10, TGF-β, etc.) and pro-angiogenic factors (bFGF, VEGF, etc.), thus terminating the inflammatory process. M2 macrophage is also known as the pro-angiogenic macrophage [26–28]. In general, macrophages facilitate the removal of apoptotic and necrotic cells and coordinate tissue repair such as angiogenesis and scar formation. In autologous tissue transplantation, M2 macrophages can benefit tissue repair [29]. In this study, our results support that SVF cells do not reduce the total number of infiltrating immune cells (macrophages) in the graft after the transplantation but increases the ratio of M2 macrophages. This may be explained by the anti-inflammatory function of SVF cells.

Premaratne et al. demonstrated that SVF therapy can reduce inflammatory factors such as TNF-α, IL-6 in chronic ischemic myocardium [11]. Donizetti-Oliveira reported that ASC treatment prevents renal disease mostly because of the inflammatory response which increases the expression of anti-inflammatory factors IL-4, IL-10 [12]. Once recruited into VEGF-expressing tissues, monocytes differentiate into M2-like macrophages and regulate both angiogenesis and vascular patterning by producing pro-angiogenic factors (including VEGF-A) and extracellular matrix (ECM) -remodelling enzymes [30]. Linde et al., concluded that the enhanced production of VEGF-A, together with IL-4 and IL-10, might promote infiltration of M2-polarized macrophages [31]. Thus, SVF cells could stimulate monocytes/macrophages to transform into angiogenic and anti-inflammatory M2 macrophages, and these M2 macrophages will improve graft survival in the later stage after adipose transplantation.

Taken together, the key finding of our present study indicates that ASC is the only cell type that survives the ischemia during the early stage of transplant, which signifies that these cells play a key role in the following regeneration progress. Therefore, increasing the retention of the ASCs in cell-assisted fat grafting appears to be of particular importantance. Recently, Gomez-Mauricio RG and his colleagues developed semipermeable membrane microcapsules labeled with Endorem (magnetocapsules) that provide mechanical and immunological immune protection to the ASCs while maintaining internal cell microenvironment while injected in vivo [32]. If this method works, the particles may also help to improve the therapeutic results in ASC-assisted fat grafting.

There are also some limitations of this study. Firstly, unisolated SVF cells in adipose tissue are surrounded by ECM which protects the cell components. However, in our study, the marked SVF cells were isolated from adipose tissue, thus the lack of ECM may not stimulate the survival condition. Secondly, this study only explored the possible functions of the ASCs in the transplant, though some researchers suggest that certain circulating cells derived from bone marrow could also be the adipose precursor cells [33,34]. Thirdly, although the nude mouse xenograft model was widely used in studies of adipose tissue/ASCs transplantation [35–37], there are still limitations. The nude mouse is a laboratory mouse from a strain with a genetic mutation that causes a deteriorated or absent thymus, resulting in an inhibited immune system due to a greatly reduced number of T cells. Nonetheless, inflammation is proved to be important in adipose regeneration, and T cells also play an important role in it [38,39]. The lack of T cell might decrease the level of the whole inflammatory reaction, although there is no further research to prove it. In summary, in our next study to further explore the regeneration of adipose tissue after transplantation, the C57BL/6J mice/green fluorescent protein (GFP) positive mice (C57BL/6J genetic background) adipose tissue exchange model could be used.

## Conclusions

This report demonstrates that the cell population which survives the ischemia after nonvascularized adipose tissue is ASCs and not the mature adipocytes nor ECs in adipose tissue. Furthermore, SVF cells could increase the ratio of the infiltrated M2 macrophages, and the immunoregulatory function of SVF cells might be beneficial to the adipose transplantation through an indirect mechanism. It might be possible to utilize these properties to enhance vascularization of autologous and engineered constructs.

## References

[1] Kaufman MR, Miller TA, Huang C, Roostaeian J, Wasson KL et al. (2007) Autologous fat transfer for facial recontouring: is there science behind the art? Plast Reconstr Surg 119: 2287-2296. doi:10.1097/01.prs.0000260712.44089.e7. PubMed: 17519732. 17519732

[2] Billings EJ, May JJ (1989) Historical review and present status of free fat graft autotransplantation in plastic and reconstructive surgery. Plast Reconstr Surg 83: 368-381. doi:10.1097/00006534-198902000-00033. PubMed: 2643129. 2643129

[3] Bircoll M (1987) Cosmetic breast augmentation utilizing autologous fat and liposuction techniques. Plast Reconstr Surg 79: 267-271. doi:10.1097/00006534-198702000-00022. PubMed: 3809274. 3809274

[4] Mackay DR, Manders EK, Saggers GC, Schenden MJ, Zaino R (1993) The fate of dermal and dermal-fat grafts. Ann Plast Surg 31: 42-46. doi:10.1097/00000637-199331010-00009. PubMed: 7689314. 7689314

[5] Aguena M, Fanganiello RD, Tissiani LA, Ishiy FA, Atique R et al. (2012) Optimization of parameters for a more efficient use of adipose-derived stem cells in regenerative medicine therapies. Stem Cells Int, 2012: 2012: 303610. PubMed: 22550502 10.1155/2012/303610 PMC3328333 22550502

[6] Mizuno H, Tobita M, Uysal AC (2012) Concise review: Adipose-derived stem cells as a novel tool for future regenerative medicine. Stem Cells 30: 804-810. doi:10.1002/stem.1076. PubMed: 22415904. 22415904

[7] van Dijk A, Naaijkens BA, Jurgens WJ, Nalliah K, Sairras S et al. (2011) Reduction of infarct size by intravenous injection of uncultured adipose derived stromal cells in a rat model is dependent on the time point of application. Stem. Cell Res 7: 219-229. 10.1016/j.scr.2011.06.003 21907165

[8] Rehman J, Traktuev D, Li J, Merfeld-Clauss S, Temm-Grove CJ et al. (2004) Secretion of angiogenic and antiapoptotic factors by human adipose stromal cells. Circulation 109: 1292-1298. doi:10.1161/01.CIR.0000121425.42966.F1. PubMed: 14993122. 14993122

[9] Sheng L, Yang M, Li H, Du Z, Yang Y et al. (2011) Transplantation of adipose stromal cells promotes neovascularization of random skin flaps. Tohoku J Exp Med 224: 229-234. doi:10.1620/tjem.224.229. PubMed: 21701129. 21701129

[10] Koh YJ, Koh BI, Kim H, Joo HJ, Jin HK et al. (2011) Stromal vascular fraction from adipose tissue forms profound vascular network through the dynamic reassembly of blood endothelial cells. Arterioscler Thromb Vasc Biol 31: 1141-1150. doi:10.1161/ATVBAHA.110.218206. PubMed: 21393582. 21393582

[11] Premaratne GU, Ma LP, Fujita M, Lin X, Bollano E et al. (2011) Stromal vascular fraction transplantation as an alternative therapy for ischemic heart failure: anti-inflammatory role. J Cardiothorac Surg 6: 43. doi:10.1186/1749-8090-6-43. PubMed: 21453457. 21453457 PMC3079611

[12] Donizetti-Oliveira C, Semedo P, Burgos-Silva M, Cenedeze MA, Malheiros DM et al. (2012) Adipose tissue-derived stem cell treatment prevents renal disease progression. Cell Transplant 21: 1727-1741. doi:10.3727/096368911X623925. PubMed: 22305061. 22305061

[13] Eto H, Suga H, Matsumoto D, Inoue K, Aoi N et al. (2009) Characterization of structure and cellular components of aspirated and excised adipose tissue. Plast Reconstr Surg 124: 1087-1097. doi:10.1097/PRS.0b013e3181b5a3f1. PubMed: 19935292. 19935292

[14] Yoshimura K, Sato K, Aoi N, Kurita M, Inoue K et al. (2008) Cell-assisted lipotransfer for facial lipoatrophy: efficacy of clinical use of adipose-derived stem cells. Dermatol Surg 34: 1178-1185. doi:10.1111/j.1524-4725.2008.34256.x. PubMed: 18513295. 18513295

[15] Eto H, Kato H, Suga H, Aoi N, Doi K et al. (2012) The fate of adipocytes after nonvascularized fat grafting: evidence of early death and replacement of adipocytes. Plast Reconstr Surg 129: 1081-1092. doi:10.1097/PRS.0b013e31824a2b19. PubMed: 22261562. 22261562

[16] Eto H, Suga H, Inoue K, Aoi N, Kato H et al. (2011) Adipose injury-associated factors mitigate hypoxia in ischemic tissues through activation of adipose-derived stem/progenitor/stromal cells and induction of angiogenesis. Am J Pathol 178: 2322-2332. doi:10.1016/j.ajpath.2011.01.032. PubMed: 21514444. 21514444 PMC3081200

[17] Planat-Benard V, Silvestre JS, Cousin B, André M, Nibbelink M et al. (2004) Plasticity of human adipose lineage cells toward endothelial cells: physiological and therapeutic perspectives. Circulation 109: 656-663. doi:10.1161/01.CIR.0000114522.38265.61. PubMed: 14734516. 14734516

[18] Kim YM, Jeon ES, Kim MR, Jho SK, Ryu SW et al. (2008) Angiotensin II-induced differentiation of adipose tissue-derived mesenchymal stem cells to smooth muscle-like cells. Int J Biochem Cell Biol 40: 2482-2491. doi:10.1016/j.biocel.2008.04.016. PubMed: 18571460. 18571460

[19] Lin G, Garcia M, Ning H, Banie L, Guo YL et al. (2008) Defining stem and progenitor cells within adipose tissue. Stem Cells Dev 17: 1053-1063. doi:10.1089/scd.2008.0117. PubMed: 18597617. 18597617 PMC2865901

[20] Cervelli V, Gentile P (2009) Use of cell fat mixed with platelet gel in progressive hemifacial atrophy. Aesthetic Plast Surg 33: 22-27. doi:10.1007/s00266-008-9223-x. PubMed: 18704559. 18704559

[21] Suga H, Matsumoto D, Eto H, Inoue K, Aoi N et al. (2009) Functional implications of CD34 expression in human adipose-derived stem/progenitor cells. Stem Cells Dev 18: 1201-1210. doi:10.1089/scd.2009.0003. PubMed: 19226222. 19226222

[22] Maumus M, Peyrafitte JA, D'Angelo R, Fournier-Wirth C, Bouloumié A et al. (2011) Native human adipose stromal cells: localization, morphology and phenotype. Int J Obes (Lond) 35: 1141-1153. doi:10.1038/ijo.2010.269. PubMed: 21266947. 21266947 PMC3172585

[23] Zimmerlin L, Donnenberg VS, Pfeifer ME, Meyer EM, Péault B et al. (2010) Stromal vascular progenitors in adult human adipose tissue. Cytometry A 77: 22-30. PubMed: 19852056. 19852056 10.1002/cyto.a.20813 PMC4148047

[24] Hollenbeck ST, Senghaas A, Komatsu I, Zhang Y, Erdmann D et al. (2012) Tissue engraftment of hypoxic-preconditioned adipose-derived stem cells improves flap viability. Wound Repair Regen 20: 872-878. doi:10.1111/j.1524-475X.2012.00854.x. PubMed: 23110692. 23110692

[25] Li P, Lu M, Nguyen MT, Bae EJ, Chapman J et al. (2010) Functional heterogeneity of CD11c-positive adipose tissue macrophages in diet-induced obese mice. J Biol Chem 285: 15333-15345. doi:10.1074/jbc.M110.100263. PubMed: 20308074. 20308074 PMC2865288

[26] Kawanishi N, Yano H, Yokogawa Y, Suzuki K (2010) Exercise training inhibits inflammation in adipose tissue via both suppression of macrophage infiltration and acceleration of phenotypic switching from M1 to M2 macrophages in high-fat-diet-induced obese mice. Exerc Immunol Rev 16: 105-118. PubMed: 20839495. 20839495

[27] Mosser DM (2003) The many faces of macrophage activation. J Leukoc Biol 73: 209-212. doi:10.1189/jlb.0602325. PubMed: 12554797. 12554797

[28] Pollard JW (2004) Tumour-educated macrophages promote tumour progression and metastasis. Nat Rev Cancer 4: 71-78. doi:10.1038/nrc1256. PubMed: 14708027. 14708027

[29] Badylak SF, Valentin JE, Ravindra AK, McCabe GP, Stewart-Akers AM (2008) Macrophage phenotype as a determinant of biologic scaffold remodeling. Tissue Eng Part A 14: 1835-1842. doi:10.1089/ten.tea.2007.0264. PubMed: 18950271. 18950271

[30] Squadrito ML, De Palma M (2011) Macrophage regulation of tumor angiogenesis: implications for cancer therapy. Mol Aspects Med 32: 123-145. doi:10.1016/j.mam.2011.04.005. PubMed: 21565215. 21565215

[31] Linde N, Lederle W, Depner S, van Rooijen N, Gutschalk CM et al. (2012) Vascular endothelial growth factor-induced skin carcinogenesis depends on recruitment and alternative activation of macrophages. J Pathol 227: 17-28. doi:10.1002/path.3989. PubMed: 22262122. 22262122

[32] Gomez-Mauricio RG, Acarregui A, Sánchez-Margallo FM, Crisóstomo V, Gallo I et al. (2013) A preliminary approach to the repair of myocardial infarction using adipose tissue-derived stem cells encapsulated in magnetic resonance-labelled alginate microspheres in a porcine model. Eur J Pharm Biopharm 84: 29-39. doi:10.1016/j.ejpb.2012.11.028. PubMed: 23266493. 23266493

[33] Lilja HE, Morrison WA, Han XL, Palmer J, Taylor C et al. (2013) An adipoinductive role of inflammation in adipose tissue engineering: key factors in the early development of engineered soft tissues. Stem Cells Dev 22: 1602-1613. doi:10.1089/scd.2012.0451. PubMed: 23231040. 23231040 PMC3653391

[34] Debels H, Galea L, Han XL, Palmer J, van Rooijen N et al. (2013) Macrophages Play a Key Role in Angiogenesis and Adipogenesis in a Mouse Tissue Engineering Model. Tissue Eng A. 10.1089/ten.tea.2013.0071 PMC3856912 23844978

[35] Rada T, Carvalho PP, Santos TC, Castro AG, Reis RL et al. (2013) Chondrogenic Potential of Two hASCs Subpopulations Loaded onto Gellan Gum Hydrogel Evaluated in a Nude Mice Model. Curr Stem Cell Res Ther 8: 357-364. doi:10.2174/1574888X113089990049. PubMed: 23755728. 23755728

[36] Fisher C, Grahovac TL, Schafer ME, Shippert RD, Marra KG et al. (2013) Comparison of harvest and processing techniques for fat grafting and adipose stem cell isolation. Plast Reconstr Surg 132: 351-361. doi:10.1097/PRS.0b013e3182958796. PubMed: 23584621. 23584621

[37] Hung MJ, Wen MC, Huang YT, Chen GD, Chou MM et al. (2013) Fascia tissue engineering with human adipose-derived stem cells in a murine model: Implications for pelvic floor reconstruction. J Formos Med Assoc. 10.1016/j.jfma.2013.04.017 23791005

[38] Nishimura S, Manabe I, Nagasaki M, Eto K, Yamashita H et al. (2009) CD8+ effector T cells contribute to macrophage recruitment and adipose tissue inflammation in obesity. Nat Med 15: 914-920. doi:10.1038/nm.1964. PubMed: 19633658. 19633658

[39] Harford KA, Reynolds CM, McGillicuddy FC, Roche HM (2011) Fats, inflammation and insulin resistance: insights to the role of macrophage and T-cell accumulation in adipose tissue. Proc Nutr Soc 70: 408-417. doi:10.1017/S0029665111000565. PubMed: 21835098. 21835098

